# Recent Advances in Pharmaceutical Cocrystals: A Focused Review of Flavonoid Cocrystals

**DOI:** 10.3390/molecules28020613

**Published:** 2023-01-06

**Authors:** Jia Xu, Qin Shi, Yanan Wang, Yong Wang, Junbo Xin, Jin Cheng, Fang Li

**Affiliations:** School of Pharmacy, Jiangsu Vocational College of Medicine, Yancheng 224005, China

**Keywords:** pharmaceutical cocrystals, flavonoids, structure–property relationships, solubility, bioavailability

## Abstract

Cocrystallization is currently an attractive technique for tailoring the physicochemical properties of active pharmaceutical ingredients (APIs). Flavonoids are a large class of natural products with a wide range of beneficial properties, including anticancer, anti-inflammatory, antiviral and antioxidant properties, which makes them extensively studied. In order to improve the properties of flavonoids, such as solubility and bioavailability, the formation of cocrystals may be a feasible strategy. This review discusses in detail the possible hydrogen bond sites in the structure of APIs and the hydrogen bonding networks in the cocrystal structures, which will be beneficial for the targeted synthesis of flavonoid cocrystals. In addition, some successful studies that favorably alter the physicochemical properties of APIs through cocrystallization with coformers are also highlighted here. In addition to improving the solubility and bioavailability of flavonoids in most cases, flavonoid cocrystals may also alter their other properties, such as anti-inflammatory activity and photoluminescence properties.

## 1. Introduction

Cocrystals are neutral crystalline single-phase materials that contain two or more discrete neutral molecules with different stoichiometry in a crystalline lattice through noncovalent interactions including hydrogen bonds, *π-π* interactions, halogen bonds and van der Waals interactions [1,2,3,4,5]. For pharmaceutical cocrystals, at least one of the coformers is an active pharmaceutical ingredient (API), and the others are pharmaceutically acceptable ingredients [6,7]. Since the crystal structure of a cocrystal is different from any starting material, its physicochemical properties may also be different. In the pharmaceutical industry, pharmaceutical cocrystals have been applied to modify the physicochemical properties of drugs, such as solubility, dissolution rate, bioavailability, hygroscopicity, compressibility, tabletability and stability [8,9,10,11,12,13,14,15,16,17]. Although some other strategies including salt formation, solvates and polymorphs have also been used to tune the physicochemical properties of drugs [18,19,20,21,22], cocrystals are much more attractive because they can alter the properties of drugs by designing supramolecular synths without changing the chemical structures of APIs. Cocrystals can alter the physicochemical properties of drugs because that crystal structures of cocrystals are different from APIs. Thus, the different interactions will have an effect on properties. For example, the cocrystal of caffeine and methyl gallate shows much better compaction properties than the coformers, because it exhibits flat sliding planes in the cocrystal’s crystal structure, which makes the compound more prone to deformation [17]. Not only that, cocrystals are mostly stable under normal conditions and can theoretically be applied to most APIs with hydrogen bond acceptors and/or donors.

**Figure 1:**
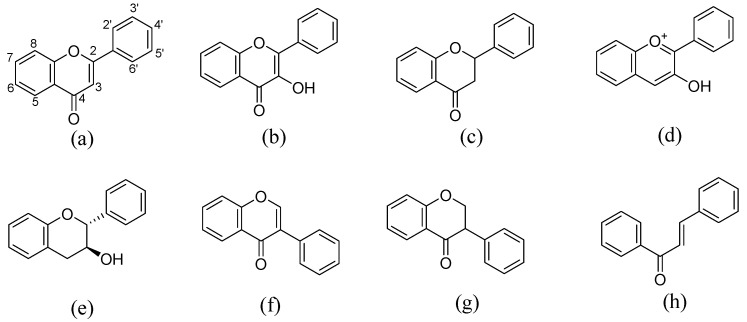
Structures of (**a**) flavone, (**b**) flavonol, (**c**) flavanone, (**d**) anthocyanidins, (**e**) flavan-3-ols, (**f**) isoflavone, (**g**) isoflavanone and (**h**) chalcone.

Flavonoids, belonging to the family of natural products with variable phenolic structures, widely exist in fruits, vegetables, bark, roots, grains, stems, tea, flowers and wine [23,24,25,26,27,28,29,30,31,32], and their basic structure consists of two phenyl rings and one heterocyclic ring [33]. Generally speaking, flavonoids can be divided into two categories, namely 2-phenylchromen and 3-phenylchromen. The first category includes flavones, flavanones, flavonols, anthocyanidins and flavan-3-ols, while the second one includes isoflavones and isoflavanones [34,35,36]. Unlike the flavonoids mentioned above, chalcone is unique in that it lacks an oxygen-heterocyclic ring but has a 3-carbon chain that acts as a bridge connecting the two phenyl rings. The molecular structures of common flavonoids are shown in Figure 1. Since Albert Szent-Gyorgyi first reported the activity of citrus peel flavonoids in preventing scurvy-related capillary hemorrhage and fragility in 1938, more biological activities have been found in flavonoids, including anticancer, anti-inflammatory, antiviral, antioxidant, antibacterial and neuroprotective activities [37,38,39,40,41,42,43,44,45,46,47]. However, their unfavorable properties such as poor bioavailability largely limit their clinical applications. Cocrystallization may be a good strategy to address this issue [48], and flavonoid cocrystals have become an ever-growing field in recent years.

In this review, we not only summarize the reported flavonoid cocrystals, but also examine and analyze the interactions present in their crystal structures to find the specific interaction types and groups that are more likely to interact with the coformers. Meanwhile, the research findings of improving the solubility and bioavailability of flavonoids by forming cocrystals are introduced. Finally, we also highlight some cases in which other properties of flavonoids are regulated through cocrystallization.

## 2. Cocrystals of Flavonoids

Cocrystals of flavonoids were first reported by Daren et al. in 2008 [49]. In the study, they described four cocrystals with certain crystal structures formed by three different flavonoids and diazobicyclooctane. Since then, over 100 cocrystals have been synthesized from more than 10 flavonoids with different coformers, of which more than 60 single crystals have been cultivated. Table 1 summarizes the subclass and number of reported flavonoid cocrystals. These APIs belong to six different flavonoid subclasses, namely flavonols, flavones, flavanones, isoflavones, chalcones and dihydrochalcones, while most APIs are flavonols and flavones, which are the two largest subclasses of flavonoids. Among them, flavonols are more likely to form cocrystals; one of the reasons is that their 3-position phenolic groups tend to form intermolecular hydrogen bonds with coformers. Additionally, quercetin, one of the most studied flavonoids, has been reported to form 60 cocrystals with 22 single crystals having been solved, and its big potential to form cocrystals may be attributed to the polyhydroxy structure. Through comparison, it can be found that 12 of the 15 flavonoids have a phenolic group at the 7-position, which may be because the 7-position phenolic group is more likely to form intermolecular hydrogen bonds with the coformers. In addition, due to the different nomenclature from other subclasses, isoliquiritigenin and phloretin, which belong to chalcone and dihydrochalcone, respectively, have a phenolic group at the 4″-position, which is equivalent to that of other flavonoids at the 7-position. Not only that, 12 of the 15 flavonoids have a phenolic group at the 4′-position, which may also be easier to form intermolecular hydrogen bonds with coformers. From a steric hindrance point of view, the 7- and 4′- positions are the two substituents that are most likely to interact with other molecules with the least steric hindrance. Thus, except that 3,6-dihydroxyflavone has two phenolic groups at the 6- and 3- positions, all other reported cocrystallized flavonoids have at least one phenolic group at the 7- or 4′- position. In addition, the toxicology of pharmaceutical cocrystals is also one of the important factors we need to consider. Therefore, the coformers are usually selected from the lists of generally regarded as safe (GRAS) and pharmaceutically accepted salt formers. Since these compounds have been previously approved by the Food and Drug Administration, utilizing them for cocrystallization can reduce preclinical burden, toxicity risk and clinical trial time.

## 3. Structures of Flavonoid Cocrystals

According to the literature, more than 60 single crystals of flavonoid cocrystals have been cultivated. Some common features can be observed from these crystal structures. Flavonoids with a phenol group at the 5-position tend to interact with their carbonyl group at the 4-position to form an intramolecular hydrogen bond, which are common in cocrystal structures containing hesperetin, genistein, baicalein, etc. However, the phenol group at the 3-positon does not interact with the neighboring carbonyl group, but dimers composed of these two groups are sometimes observed in the structures of flavonoid cocrystals, such as quercetin-4,4′-bipyridine [55], fisetin-caffeine [63], etc. The coformers of flavonoid cocrystals also have some structural characteristics. Flavonoids tend to form cocrystals with N-containing heterocyclic compounds such as nicotinamide, isonicotinamide, theophylline, caffeine, 4,4′-bipyridine, proline, etc. However, among these compounds, nicotinamide and isonicotinamide are most likely to be selected as coformers, since the N-atom on their pyridine ring is a good hydrogen bond acceptor and tends to interact with the phenol group of flavonoids. This feature can also be observed in the structures of most flavonoid cocrystals whose coformers contain a nitrogen heterocylic ring, such as isoliquritigenin–nicotinamide [78], genistein–caffeine [77], etc. Furthermore, amide groups containing both hydrogen bond donors and acceptors tend to interact with the phenol groups of flavonoids, which can be observed in the structures of baicalein–isonicotinamide [69], fisetin–nicotinamide [63], etc. Several typical crystal structures of flavonoid cocrystals with different features are analyzed below.

### 3.1. Quercetin–Isonicotinamide Cocrystal

**Figure 2:**
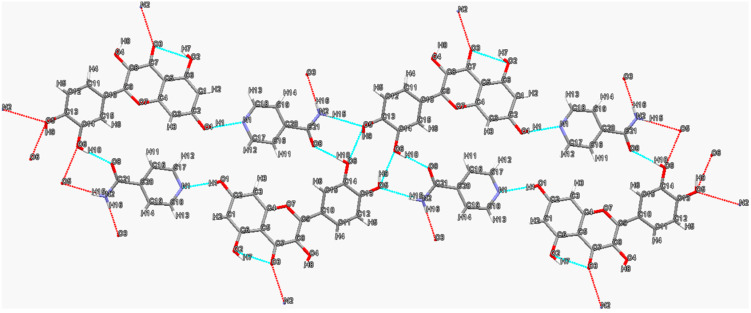
Two-dimensional hydrogen bonding network in quercetin–isonicotinamide cocrystal. Hydrogen bonds are indicated by blue dashed lines. Red dashed lines indicate further interactions with other molecules not shown.

The two-dimensional hydrogen bond network in the quercetin (QUE)–isonicotinamide (INM) cocrystal [52] is shown in Figure 2. Structural analysis reveals that the centrosymmetric tetramer assembled by two QUE molecules and two INM molecules is the basic unit of this cocrystal. First, two quercetin molecules form a dimeric unit via the $R_{2}^{2}$(10) supramolecular homosynthon by means of O-H⋯O hydrogen bonding interactions (O5-H9···O6, 2.01 Å, 150°). Subsequently, the QUE homodimeric units are further linked with two INM molecules via N2-H15·O5 (2.15 Å, 171°) and O6-H10···O8 (1.78 Å, 172°) hydrogen bonds to form a tetramer. Finally, the tetrameric motif is extended to form a two-dimensional (2D) network through O1-H1···N1 (1.86 Å, 167°) and N2-H16···O3 (2.17 Å, 178°) hydrogen bonds. Likewise, tetramers consisting of two drug molecules and two coformer molecules are also commonly found in the structures of other flavonoid cocrystals, such as fisetin–nicotinamide [63], fisetin–isonicotinamide [66] and luteolin–isonicotinamide [66] cocrystals. In fact, all reported flavonoid cocrystal tetramers have been assembled in the similar pattern so far. In these assemblies, the $R_{2}^{2}$(10) “homo-dimer” formed by two flavonoid molecules is further linked by nicotinamide or isonicotinamide molecules to form the $R_{3}^{3}$(8) graph set. This unique tetrameric motif may depend on the common structural features of these flavonoids. For example, in the structures of fisetin, luteolin and quercetin, the two phenolic groups located at the 3′- and 4′-positions of the ortho-position of the benzene ring have the smallest steric hindrance and are more likely to interact with other flavonoid molecules to form dimers.

### 3.2. Quercetin–Isonicotinic Acid Monohydrate

**Figure 3:**
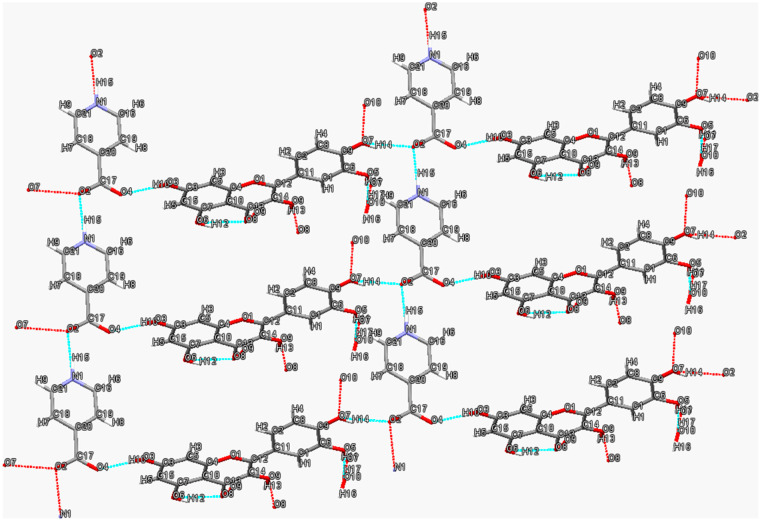
Two-dimensional hydrogen bonding network in quercetin-isonicotinic acid monohydrate. Hydrogen bonds are represented by blue dashed lines. Red dashed lines indicate further interactions with other molecules not shown.

The two-dimensional hydrogen bonding network in quercetin (QUE)-isonicotinic acid (INA) monohydrate [57] is shown in Figure 3. Apparently, the carboxylate moieties of INA are H-bonded to the hydroxyl moieties on either side of the chains O7-H14···O2 (1.98 Å, 147°) and O3-H10···O4 (1.81 Å, 171°) of the QUE molecules, while the water molecules connect adjacent quercetin molecules via O10-H17···O7 (1.83 Å, 173°) and O5-H11···O10 (1.80 Å, 170°) hydrogen bonds (another quercetin molecule not shown in the figure). The crystal structure of the 1:1 cocrystal monohydrate of QUE and INA contains INA zwitterions that form parallel chains through a N1-H15···O2 (1.57 Å, 170.2°) hydrogen bond, which is supported by the C-N-C angle of 121.7°and C-O bond distances of 1.244 A° and 1.263 A°.

### 3.3. Baicalein-Nicotinamide Cocrystal

**Figure 4:**
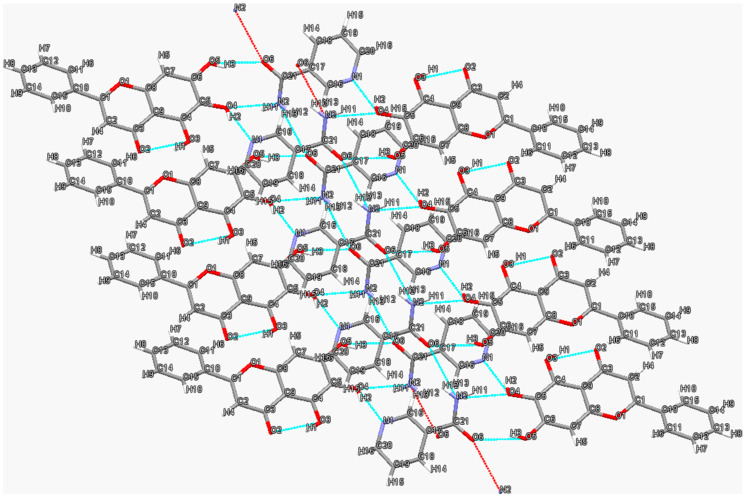
Two-dimensional hydrogen bonding network in baicalein-nicotinamide cocrystal. Hydrogen bonds are represented by blue dashed lines. Red dashed lines indicate further interactions with other molecules not shown.

The two-dimensional hydrogen bond network in the baicalein–nicotinamide cocrystal [68] is shown in Figure 4. Nicotinamide molecules form two types of parallel molecular chains in converse ordinations through N2-H12···O6 (2.23 Å, 145°) hydrogen bonds, and their amide moieties interact with the ortho-phenyl groups of adjacent baicalein molecules via O5-H3···O6 (1.94 Å, 154°) and N2-H11···O4 (2.33 Å, 121°) hydrogen bonds to form heterodimers, while their pyridine nitrogen atoms interact with the neighboring baicalein molecules in the other direction through O4-H2···N1 (1.91 Å, 155°) hydrogen bonds. Every baicalein molecule is connected with two nicotinamide molecules in different chains via O5-H3···O6 (1.94 Å, 154°), N2-H11···O4 (2.33 Å, 121°) and O4-H2···N1 (1.91 Å, 155°) hydrogen bonds, thus forming a tetramer consisting of two baicalein molecules and two nicotinamide molecules. The tetramer extends along the direction parallel to nicotinamide chains to form the hydrogen-bonded networks.

### 3.4. Isoliquiritigenin–Isonicotinamide Cocrystal

**Figure 5:**
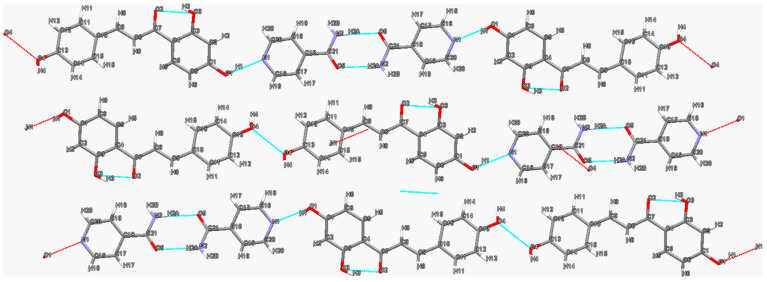
Two-dimensional hydrogen bonding network in isoliquiritigenin–isonicotinamide cocrystal. Hydrogen bonds are represented by blue dashed lines. Red dashed lines indicate further interactions with other molecules not shown.

The two-dimensional hydrogen bond network of the isoliquiritigenin–isonicotinamide (ISL-INM) cocrystal [78] is displayed in Figure 5. Obviously, ISL and INM molecules are assembled into a sheet structure in their cocrystal. The amide moiety of two adjacent INM molecules is connected by two N2-H2A···O5 (2.03 Å, 172°) hydrogen bonds to form an $R_{2}^{2}$(8) homodimer, which is connected to the neighboring ISL molecules through the O1-H1···N1 (1.89 Å, 162°) hydrogen bond. The central INM dimer is capped by a flavonoid molecule at each end, forming a 0-D motif. Then, the 0-D motifs interact with each other through N2-H2B···O2 (2.25 Å, 168°) and C14-H14···O1 (2.56 Å, 156°) hydrogen bonds to form a 2D sheet structure. Meanwhile, the oxygen atoms (O4) of phenol groups on the adjacent ISL molecules face each other in a close-packed arrangement, which helps to arrange 0-D motifs into a line [80]. Different from other packing types (such as serrated layer), the molecules in the ISL-INM cocrystal are packed into flat layers with relatively large spacing. Under the influence of shear stress, it is easier to slide between neighboring layers, which may lead to higher plasticity and better tableting performance [15,81].

## 4. Functions of Flavonoid Cocrystals

### 4.1. Improving Solubility and Bioavailability

Flavonoids are a large family of natural products with a variety of biological activities, including anticancer, anti-inflammatory, antiviral, antioxidant, antibacterial and neuroprotective activities [37,38,39,40,41,42,43,44,45,46,47]. However, the solubility and bioavailability of most flavonoids are poor, which largely limits the further exploitation of flavonoids as drugs [82]. The cocrystallization of flavonoids and soluble coformers may solve these problems, and several cases are discussed in detail below.

**Figure 6:**
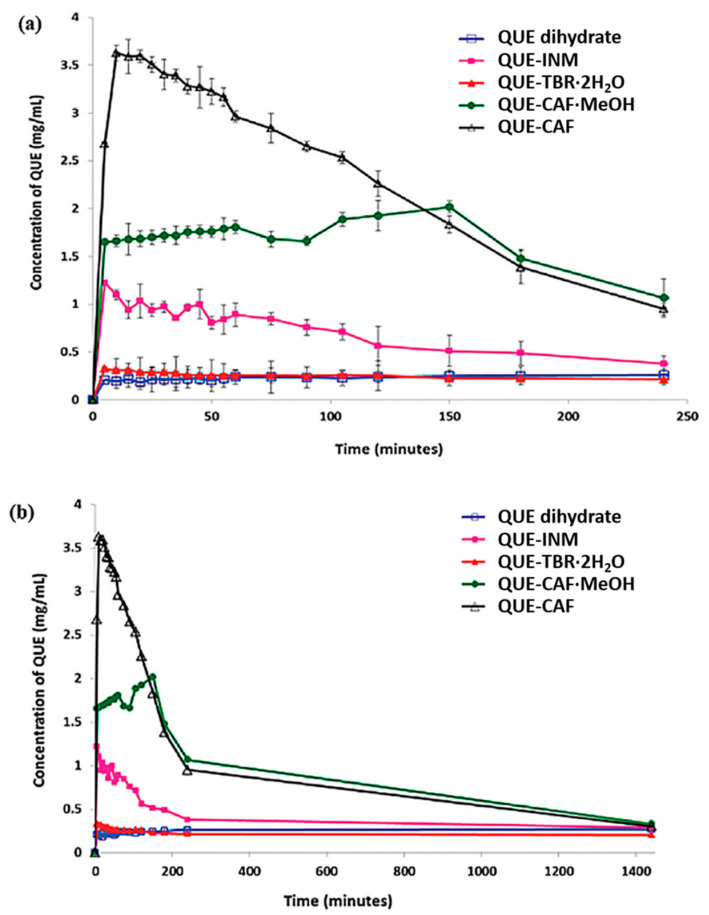
Dissolution profiles of QUE dihydrate and QUE cocrystals in 1:1 methanol/water mixture during (**a**) the first 4 h and (**b**) 24 h. Adapted from [52] with permission. Copyright © 2011 American Chemical Society.

**Figure 7:**
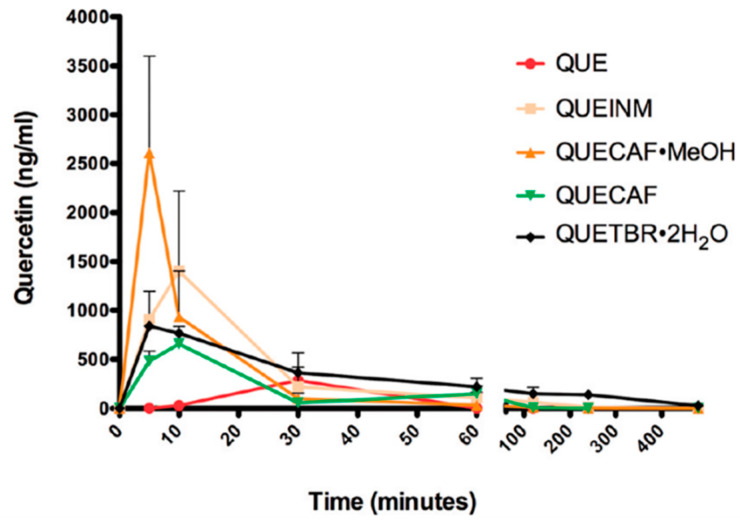
Pharmacokinetic profiles of QUE preparations (mean plasma concentration + SD, *n* = 3). Statistical significances were achieved between QUE-INM and QUE at t = 10 min (*p* < 0.01) and between QUE-CAF·MeOH and QUE at t = 5 min (*p* < 0.001), respectively. Adapted from [52] with permission. Copyright © 2011 American Chemical Society.

As one of the most abundant flavonoids in the plant kingdom, quercetin (QUE) has numerous therapeutic bioactivities in vitro such as antioxidant, metal chelating, antiviral, bacteriostatic, anticarcinogenic and cardioprotective activities [83,84,85,86,87,88]. However, due to its low solubility and poor bioavailability, its pure form has limited efficacy in vivo [89,90,91,92,93,94]. Smith et al. [52] studied the solubility and bioavailability of four cocrystals formed by quercetin and three different coformers, including isonicotinamide, theobromine and caffeine. The dissolution curves of four cocrystals (quercetin–isonicotinamide (QUE-INM), quercetin–theobromine dehydrate (QUE-TBR·2H_2_O), quercetin–caffeine (QUE-CAF) and quercetin–caffeine monomethanolate (QUE-CAF·MeOH)) and QUE dihydrate in 50% methanol–water (*v*/*v*) are shown in Figure 6. It is not difficult to find that each of these cocrystals exhibit superior solubility to quercetin dihydrate. For example, the solubility of QUE dihydrate was 0.267 mg/mL, while the maximum solubilities of quercetin–caffeine, quercetin–caffeine monomethanolate, quercetin–isonicotinamide and quercetin–theobromine dehydrate were 3.627, 2.018, 1.22 and 0.326 mg/mL, respectively. Among these cocrystals, the concentration of quercetin dihydrate in the quercetin–caffeine cocrystal is the highest, and its solubility has increased about 13 times. It is hypothesized that an improvement in the solubility of quercetin will translate into the enhancement of its pharmacokinetic behavior, and the experimental results are shown in Figure 7. As expected, these cocrystals increased the absorption of quercetin in rats by up to 10 times in comparison to quercetin dihydrate.

**Figure 8:**
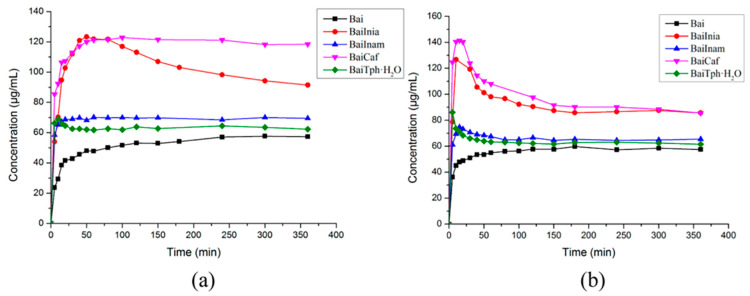
Powder dissolution profiles of Bai and its four cocrystals (BaiInia (baicalein–isoniazide), BaiInam (baicalein–isonicotinamide), BaiCaf (baicalein–caffeine) and BaiTph·H_2_O (baicalein–theophylline monohydrate)) in (**a**) pH 2.0 and (**b**) pH 4.5 buffer solutions. Adapted from [69] with permission. Copyright © 2017 American Chemical Society.

**Figure 9:**
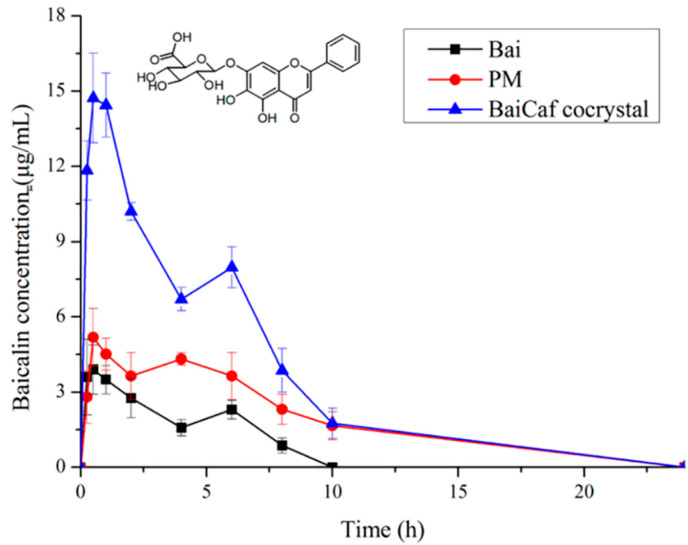
Plasma BG concentration–time curves of the crystalline Bai, PM and BaiCaf cocrystal (data are expressed as means ± SD, *n* = 6). Adapted from [69] with permission. Copyright © 2017 American Chemical Society.

As an important bioactive flavonoid compound isolated from the root of *Scutellaria baicalensis*, baicalein (Bai) has anti-inflammatory, anticancer, anti-HIV, anti-adipogenic and antibacterial activities [95,96,97,98,99,100,101]. Not only that, but it is also included in the *Chinese Pharmacopoeia* as a medication for treating fever, upper respiratory tract infection and sore throat [102]. However, the application of baicalein in the pharmaceutical field is limited, largely owing to its poor water solubility and low bioavailability [103,104]. Cocrystallization may be an effective way to address the above problems. Zhu et al., reported that baicalein–nicotinamide (BaiNam) cocrystals increased the solubility of baicalein by 50–100% in the pH range of 3.6 to 6.8. In addition, a much larger apparent solubility was also shown in baicalein–caffeine (BaiCaf) and baicalein–isonicotinamide (BaiInam) cocrystals (Figure 8) [69]. In the buffer solutions of pH 2.0 and pH 4.5, the baicalein–caffeine cocrystal resulted in the most significant solubility improvement, which was about 2.5-fold and 1.5-fold that of pure baicalin, respectively [67]. Meanwhile, the maximum solubility of the baicalein–isonicotinamide cocrystal is similar to that of the baicalin–caffeine cocrystal. As the increase in drug solubility may improve its bioavailability, Zhu et al. studied the bioavailability of the baicalein–caffeine cocrystal, a baicalein–caffeine physical mixture and pure baicalin in rats to confirm this [69]. Since baicalein-7-O-glucuronide (BG) is the main active metabolite of Bai and the mainly existing form in plasma, BG was provided for statistical comparison and bioavailability calculation. As shown in Figure 9, The *C_max_* and AUC_0–24h_ of the baicalein–caffeine cocrystal were 2.35-fold and 4.14-fold higher than those of pure baicalin (Bai), respectively, which were also significantly higher than those of their physical mixture (PM).

**Figure 10:**
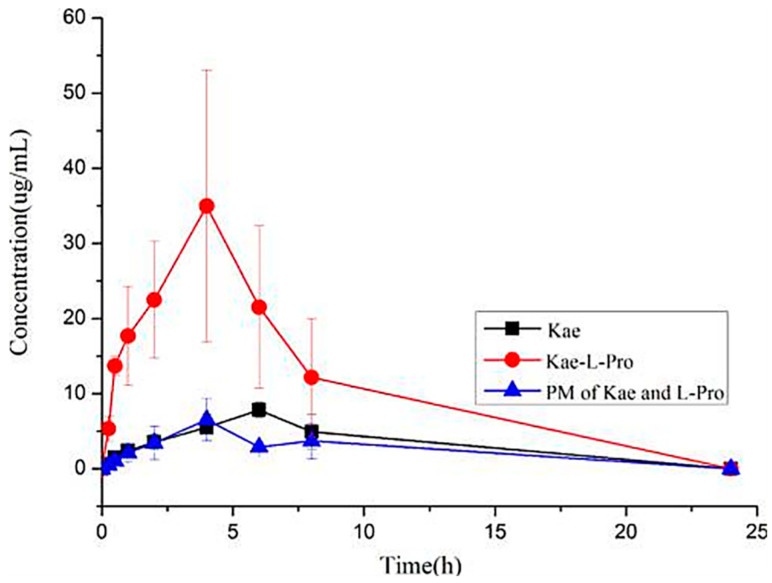
Pharmacokinetic profiles of Kae-3-o-glucosylside after administration of kaempferol (Kae), kaempferol-L-proline (Kae-L-Pro) and physical mixture (PM) of Kae and L-Pro (mean plasma concentration versus time). Data are expressed as means ± SD, *n* = 6. Adapted from [58] with permission. Copyright © 2016 American Chemical Society.

In addition, kaempferol with tetrahydroxyflavone structure is one of the most common aglycone flavonoids, which exists in various parts of plants in the form of glycosides, including seeds, leaves, fruits, flowers and even vegetables. It has been proven that kaempferol and its glycosylated derivatives have a variety of pharmacological activities, such as osteoprotective, anticancer, neuroprotective, anti-inflammatory, antidiabetic, antioxidant, antimicrobial, chemo-preventive and therapeutic activities [105,106,107,108,109,110,111]. However, like other flavonoids mentioned above, the solubility of kaempferol in water is very low, which leads to poor absorption in vivo [94,112,113]. Recently, a kaempferol-L-proline cocrystal was synthesized, and its solubility and bioavailability are higher than those of pure kaempferol [58]. The dissolution experiment of the powders in a 0.5% Tween 80 system showed that the maximum solubility of the kaempferol-L-proline cocrystal was about 270% higher than that of pure kaempferol. Meanwhile, the pharmacokinetic curves of kaempferol (Kae), the kaempferol-L-proline (Kae-L-Pro) cocrystal, and a physical mixture (PM) of kaempferol and L-proline are presented in Figure 10. As the main metabolite of Kae in blood, the pharmacokinetic parameters of Kae-3-O-glucoside were provided and analyzed. The results showed that the pharmacokinetic curve of the Kae-L-Pro cocrystal was improved compared with the pure Kae component and corresponding physical mixture, and its *C_max_* and AUC_0–24h_ were 369% and 351% higher than those of the pure Kae, respectively.

**Figure 11:**
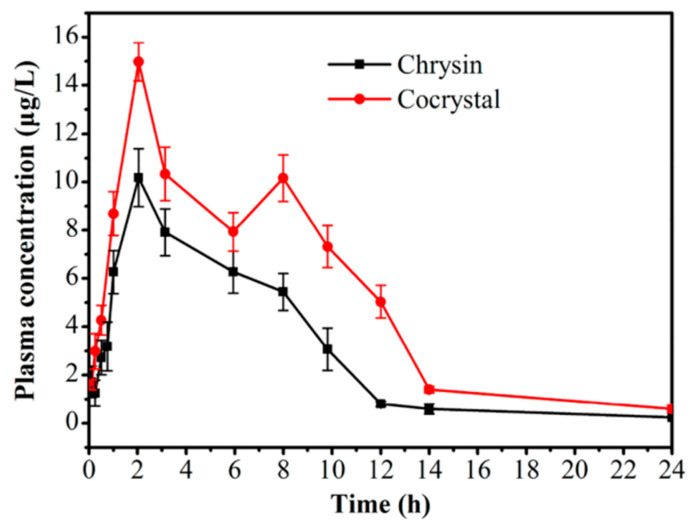
Mean plasma concentrations versus time profiles of chrysin and cocrystal. Adapted from [71] with permission. Copyright © 2018 American Chemical Society.

Chrysin, isolated from various plants such as the blue passion flower (*Passiflora caerulea* L.), is a flavonoid compound with a variety of pharmacological activities including antidiabetic, anti-inflammatory, and antitumor activities [114,115,116]. Sa et al., reported a novel salt cocrystal of chrysin (ChrH) and berberine (BerbOH) [71], which is also a new drug−drug cocrystal based on two natural products. An in vivo bioavailability study on pure chrysin and chrysin in the form of the cocrystal was performed, and the mean plasma concentrations of chrysin in the two forms versus time profiles are shown in Figure 11. The results show that the chrysin cocrystal has higher *C_max_* and AUC than pure chrysin. According to the AUC_0−24h_ results, the relative bioavailability of the chrysin cocrystal is about 1.7 times of that of pure chrysin. Although the improvement of *C_max_* and AUC is modest, this work provides a new strategy for the design of drug−drug cocrystals based on alkaloids and flavonoids through charge-assisted strong hydrogen bonding interactions.

### 4.2. Optimizing Other Properties

The cocrystallization of flavonoids can not only improve the solubility and bioavailability of APIs, but also adjust many other properties such as pharmacodynamic properties, photoluminescent properties, etc.

#### 4.2.1. Improving Pharmacodynamic Response

**Figure 12:**
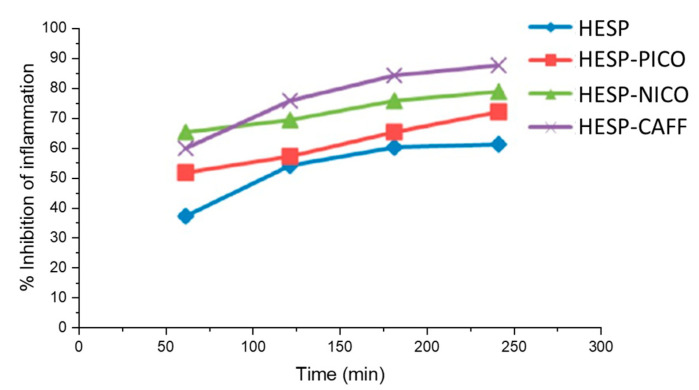
Percent inhibition of inflammation of HESP, HESP-PICO, HESP-NICO and HESP-CAFF. Adapted from [72] with permission. Copyright © 2017 American Chemical Society.

Hesperetin, commonly found in citrus fruits, is a powerful antioxidant molecule and belongs to dihydroflavonoids. It also exhibits antiplatelet, anti-inflammatory, antiviral and antibacterial effects, as well as prominent protective effects on carcinoid, lung, breast and colon cancers [117,118,119,120,121,122,123,124]. In order to evaluate the pharmacodynamic differences between hesperidin and its cocrystals, Kunal et al. studied their anti-inflammatory activity [72], and the percent inhibitions of inflammation of hesperetin (HESP), the hesperetin–picolinic acid cocrystal (HESP-PICO), the hesperetin–nicotinamide cocrystal (HESP-NICO) and the hesperetin–caffeine cocrystal (HESP-CAFF) are shown in Figure 12. Obviously, the inflammation inhibitory effect of pure hesperetin was weaker than its cocrystals at all time points, and all three cocrystals exhibited improved anti-inflammatory activity. After 240 min of carrageenan injection, all compounds generally reached the maximum inflammation inhibition percentage. At this moment, compared with the anti-inflammatory inhibition rate of 60% of pure hesperetin, HESP-CAFF showed the strongest anti-inflammatory activity, with an inflammation inhibition rate of 87%, while HESP-NICO and HESP-PICO also showed better anti-inflammatory activity, with inhibition rates of 79% and 72%, respectively. These impressive data indicate that cocrystals have a clear advantage over the drug itself in achieving the desired pharmacological response.

**Figure 13:**
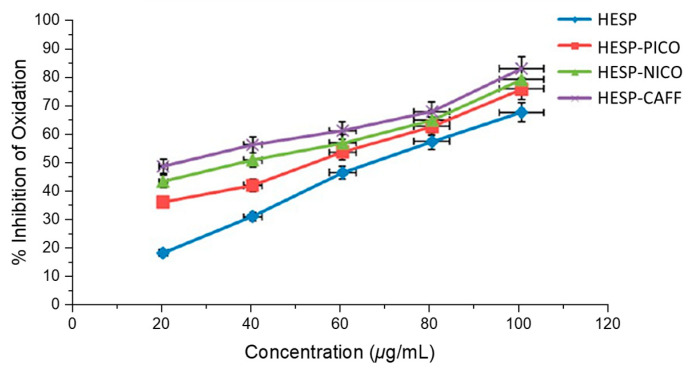
Percentage inhibition of oxidation of DPPH radical by hesperidin and the cocrystals. Adapted from [72] with permission. Copyright © 2017 American Chemical Society.

**Figure 14:**
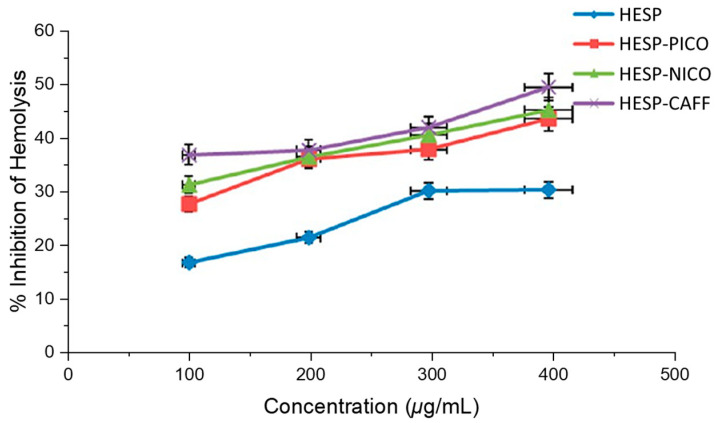
Antihemolytic activity represented as the percentage inhibition of hemolysis by hesperidin and the cocrystals. Adapted from [72] with permission. Copyright © 2017 American Chemical Society.

In addition, Kunal et al. also studied the antioxidant and antihemolytic activities of hesperetin cocrystals [72]. As shown in Figure 13, compared with pure hesperidin, the antioxidant activity of hesperetin cocrystals measured by the oxidation inhibition percentage of the 1,1-diphenyl-2-picryl hydroxyl (DPPH) free radical increased, indicating that the activity of HESP-CAFF increased by nearly 50%, the activity of HESP-NICO increased by about 30% and the activity of HESP-PICO increased by 20%. Figure 14 lucidly depicts that compared with the cocrystals, hesperidin has a much lower inhibitory effect on the hemolysis of rat red blood cells (RBCs). On the average of all tested concentrations, the hemolysis rate of rat RBCs was significantly reduced, with a maximum 60% decrease by HESP-CAFF, followed by a nearly 40% decrease by HESP- NICO and about 30% by HESP-PICO, over that of pure hesperidin.

#### 4.2.2. Tuning Photoluminescent Properties

**Figure 15:**
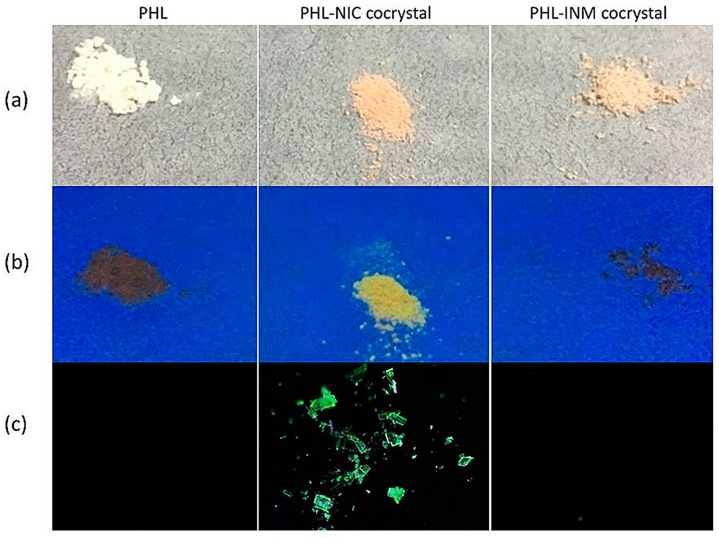
Photographs of solid-state cocrystal samples (from left to right: PHL, PHL-NIC cocrystal and PHL-INM cocrystal): (**a**) the powder samples under daylight; (**b**) the powder samples under UV (365 nm) lamp; (**c**) the single crystal samples under UV (365 nm) observed by polarized microscope. Adapted from [79] with permission. Copyright © 2019 American Chemical Society.

Phloretin (PHL), extracted from the pericarp and velamen of apples or pears, is a dihydrochalcone flavonoid. It not only has many pharmacological activities including antioxidant, anticancer and anti-inflammatory effects, but also can suppress the growth, virulence and biofilm formation of Gram-negative and Gram-positive bacteria [125,126,127,128,129,130,131,132]. Recently, in order to improve the solubility of phloretin, Huang et al. synthesized phloretin–nicotinamide (PHL-NIC) and phloretin–isonicotinamide (PHL-INM) cocrystals, and observed that phloretin, the PHL-NIC cocrystal and the PHL-INM cocrystal have apparently different photoluminescent properties [79]. As shown in Figure 15, under a 365 nm UV lamp, the PHL-NIC cocrystal exhibited strong yellowish-green fluorescence, while PHL and the PHL-INM cocrystal showed almost no fluorescence under the same condition. This result indicates that the introduction of the NIC coformer can significantly affect the photoluminescent properties of phloretin, while the introduction of the INM coformer cannot. The different photoluminescence properties of these two cocrystals may be attributed to the varied intermolecular interactions and stacking arrangements in their structures. From the perspective of cocrystal structure, compared to the sheet (planar) structure of the PHL-INM cocrystal with a shorter ring centroid−centroid (Cg-Cg) distance, the zigzag packing of the PHL-NIC cocrystal with a longer ring centroid−centroid (Cg-Cg) distance may enhance the emission of the PHL-NIC cocrystal in the solid state, resulting in high luminescent property. Additionally, the Hirshfeld surface analysis results of PHL molecules on PHL-NIC and PHL-INM cocrystals also quantitatively support this conclusion. The *π-π* interaction of the PHL-NIC cocrystal is 10.5%, which is lower than that of the PHL-INM cocrystal (12.5%). These results imply that the photoluminescence properties of flavonoid cocrystals can be tuned by the introduction of coformers.

## 5. Conclusions

Pharmaceutical cocrystals are currently a rapidly developing field, because they can favorably alter the physicochemical properties of APIs. Recently, benefiting from the polyphenolic structure, cocrystallization has become an effective method in improving the properties of flavonoids. In this review, we summarized the cocrystals synthesized from different flavonoids and coformers and discussed in detail that phenolic groups tend to form intermolecular hydrogen bonds with the coformers. On this basis, we presumed that flavonoids with a phenolic group at the 7-position or 4′-position are more likely to form cocrystals and discussed the different intermolecular and intramolecular interactions in their solid forms by analyzing the crystal structures of some typical flavonoid cocrystals. The tetramer composed of two flavonoid molecules and two nicotinamide or isonicotinamide molecules, which exists in the crystal structures of quercetin–isonicotinamide and fisetin–nicotinamide cocrystals, is the most typical arrangement. In most cases, the purpose of synthesizing flavonoid cocrystals is to improve solubility and bioavailability. Therefore, it is preferable to select the coformers with high solubility (e.g., nicotinamide and isonicotinamide) in the GRAS list. In addition, the cocrystallization of flavonoids may also alter other properties. Flavonoid cocrystals have a good prospect in clinical translation, and the analysis of possible hydrogen bond sites and hydrogen bond networks in this review is helpful for the targeted synthesis of flavonoid cocrystals.

## Figures and Tables

**Table 1:** Summary of reported flavonoid cocrystals.

Flavonoids	Structures	Subclass	Number of Cocrystals Reported ^1^	References
Daidzein	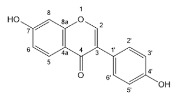	Isoflavones	0/1	[50]
Quercetin	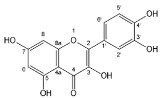	Flavonols	22/60	[49,51,52,53,54,55,56,57,58,59]
Myricetin	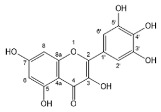	Flavonols	4/8	[55,60,61,62,63,64,65]
Fisetin	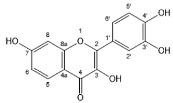	Flavonols	4/4	[63,66]
Kaempferol	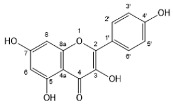	Flavonols	2/2	[55,58]
3,6-dihydroxyflavone	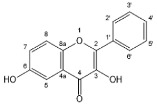	Flavonols	2/2	[49]
Baicalein	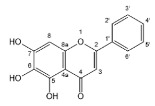	Flavones	6/8	[58,67,68,69]
Chrysin	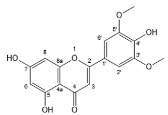	Flavones	4/4	[58,70,71]
Luteolin	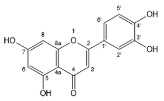	Flavones	3/3	[58,66]
Apigenin	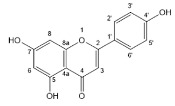	Flavones	0/1	[50]
Hesperetin	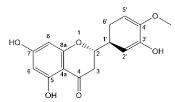	Flavanones	5/5	[57,72]
Naringenin	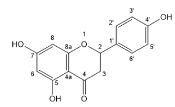	Flavanones	7/9	[49,73,74]
Genistein	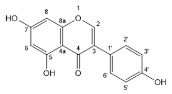	Isoflavones	5/5	[58,66,75,76,77]
Isoliquiritigenin	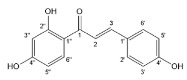	Chalcones	2/2	[78]
Phloretin	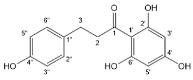	Dihydrochalcones	2/2	[79]

^1^ The number of reported cocrystals with single crystals/the total number of reported cocrystals.

## Data Availability

Not applicable.
